# The Clinical and Cellular Impact of RBMS2 on the Progression and Prognosis of Kidney Renal Clear Cell Carcinoma

**DOI:** 10.1155/2023/5512781

**Published:** 2023-11-28

**Authors:** Zhixiang Gao, Shouren Fan

**Affiliations:** ^1^Pathology Department of the Second Hospital of Tianjin Medical University, Tianjin 300211, China; ^2^Pathology Department of the Second People's Hospital of Binzhou, Binzhou 256800, China

## Abstract

This research delves into the implications of the RNA binding motif, single stranded interacting protein 2 (RBMS2)—a gene associated with tumor-suppressing functions—in the context of kidney renal clear cell carcinoma (ccRCC). Through meticulous exploration of online databases, we have identified a negative association between RBMS2 expression and adverse clinico-pathological features, such as advanced TNM stage. Furthermore, our findings indicate that RBMS2 acts as a prognostic predictor for clinical outcomes in ccRCC, evidenced by both univariate and multivariate analyses. Cellular assays have corroborated these findings, revealing that an overexpression of RBMS2 curtails ccRCC cell proliferation and migration. Additionally, our research has unearthed links between RBMS2 and immune infiltration within the ccRCC tumor microenvironment. Collectively, our results underscore the tumor-inhibiting role of RBMS2 in ccRCC and spotlight its potential as a prognostic marker and therapeutic intervention target.

## 1. Introduction

RNA binding motif, single stranded interacting protein 2 (RBMS2), a gene commonly linked with tumor-suppressive properties, is the focus of our study. Positioned on chromosome 12q13.3 [1], RBMS2 plays a critical role in the regulation of transcription and alternative splicing, both key cellular processes related to cell proliferation and programmed cell death or apoptosis. Alterations or disruptions in the normal functioning of RBMS2 have been found to be associated with the development of various types of cancers, underlining its crucial role in maintaining the delicate balance of cellular homeostasis [2].

Investigations into different types of cancers such as lung cancer, gastric cancer, and hepatocellular carcinoma have reported a downregulation of RBMS2. This diminished expression of RBMS2 is seen to contribute to the initiation and progression of these cancers, thereby establishing a link between RBMS2 downregulation and oncogenesis [3]. However, the role of RBMS2 in kidney renal clear cell carcinoma (ccRCC), which is the most prevalent subtype of kidney cancer, has not been thoroughly explored.

The primary objective of this study is to illuminate the role of RBMS2 in ccRCC and to establish its clinical and cellular implications. This is achieved by investigating the relationship between the expression of RBMS2 and the clinico-pathological characteristics of patients suffering from ccRCC. We delve into the prognostic potential of RBMS2 and its impact on the growth of ccRCC cells. An additional layer of complexity is added by the tumor microenvironment, which plays a significant role in the progression of the disease. In this context, we analyze the relationship between RBMS2 expression and immune infiltration in the tumor microenvironment of ccRCC.

The tumor microenvironment, a complex and dynamic entity, has a profound impact on the development and progression of cancer [4]. It consists of various immune cells, stromal cells, and extracellular matrix components and is known to be involved in immune surveillance, tumor progression, and therapeutic response [5]. Recent studies have started to unravel the complex interplay between cancer cells and their microenvironment, revealing that cancer cells can manipulate their surroundings to promote tumor growth, invasion, and resistance to therapy [6]. In the context of ccRCC, the tumor microenvironment has been found to be highly immunogenic, with a high degree of immune cell infiltration [7]. However, despite the immunogenic nature of ccRCC, immune checkpoint inhibitors, which are designed to reactivate the antitumor immune response, have only shown limited efficacy in this cancer type. This suggests that there are still unknown mechanisms through which ccRCC can evade the immune system and highlights the need for further research in this area.

In this study, we also shed light on these issues by investigating the role of RBMS2 in ccRCC and its relationship with the tumor microenvironment. We hope that our findings will not only contribute to the understanding of ccRCC pathogenesis but also open new avenues for the development of more effective therapeutic strategies for this devastating disease.

## 2. Materials and Methods

### 2.1. Online Database

We harnessed the potential of the Cancer Genome Atlas (TCGA) database, a comprehensive resource housing genomic and clinical data from thousands of cancer patients [8]. The ccRCC dataset from TCGA, which includes gene expression data and associated clinico-pathological information, served as the foundation for our investigation into the role of RBMS2 in ccRCC.

### 2.2. Immunohistochemical (IHC) Staining

IHC was utilized in this study to evaluate the protein expression of RBMS2 in ccRCC and adjacent nontumorous tissue samples. The process commenced with the preparation of 4 *μ*m thick tissue sections, which underwent a sequence of treatments involving deparaffinization and rehydration utilizing successive xylene and ethanol immersions. Subsequently, heat-induced epitope retrieval was conducted by subjecting the samples to antigen retrieval, employing citrate buffer (pH 6.0) within a microwave oven. To inhibit any inherent peroxidase activity, a 3% hydrogen peroxide solution was applied for a duration of 10 minutes, followed by incubation with bovine serum albumin (BSA). The tissue sections were subsequently subjected to an overnight incubation at 4°C, employing a primary antibody specific to RBMS2 at a 1 : 200 dilution. Afterward, PBS was used to thoroughly rinse the sections, and they were then exposed to a secondary antibody, conjugated with horseradish peroxidase (HRP), for an hour at room temperature. Detection of the antigen-antibody complex was facilitated by employing a 3,3′-diaminobenzidine (DAB) substrate solution, yielding a brown-colored precipitate indicative of antigen localization. Hematoxylin counterstaining was subsequently applied to facilitate the visualization of cell nuclei. Negative controls were integrated into the procedure by omitting the primary antibody.

Following the staining process, the tissue sections underwent dehydration via a series of ethanol washes and clearing with xylene. Ultimately, the slides were mounted with a coverslip utilizing mounting medium. The stained tissue sections were subsequently examined to evaluate the extent of RBMS2 protein expression.

### 2.3. Cell Culture and Transfection

Cellular experiments were performed to validate the findings from the database analysis. We used two ccRCC cell lines, 786-O and Caki-2, for these experiments. Transient transfection techniques were employed to overexpress pcDNA3.1-vector or pcDNA3.1-RBMS2 in these cell lines by Lipo3000 reagent, allowing us to observe the impact of RBMS2 on ccRCC cell growth [9]. The overexpression plasmids were synthesized in GenePharma (Shanghai, China). Western blotting was used to assess the transfection efficacy.

### 2.4. Proliferation Assay

We utilized the CCK-8 assay to assess the proliferation capacity of the ccRCC cell lines following RBMS2 overexpression based on the manufacturer's instructions as previously reported.

### 2.5. Migration Assay by Transwell

The transwell migration assay was used to evaluate the impact of RBMS2 overexpression on the migration ability of the ccRCC cell lines. Cells (2 × 10^5^) were suspended in 100 *μ*l serum-free medium at a density of 10^5^/ml and seeded into the upper chamber. The bottom wells were filled with 600 *μ*l of 10% FBS medium. Cells that had migrated or invaded to the lower surface of the membrane were fixed with methanol and glacial acetic acid and then stained with 20% Giemsaand. Five random fields were chosen to count migrated cell numbers.

## 3. Statistics

Statistical analyses were performed using R software. All data are presented as mean ± SD. The correlation between RBMS2 expression and clinico-pathological characteristics was assessed using the chi-square test. Survival analysis was conducted using the Kaplan–Meier method and the Cox regression model. Cellular experimental data were tested by Student's *t*-test. *P* < 0.05 was considered statistically significant.

## 4. Ethics Statement

This study was conducted in accordance with the Declaration of Helsinki. Since the study used publicly available data and in vitro experiments, it did not involve human participants or animals and thus did not require ethical approval.

## 5. Results

### 5.1. Interplay between RBMS2 Expression and Clinico-Pathological Traits in ccRCC Patients

**Figure 1:**
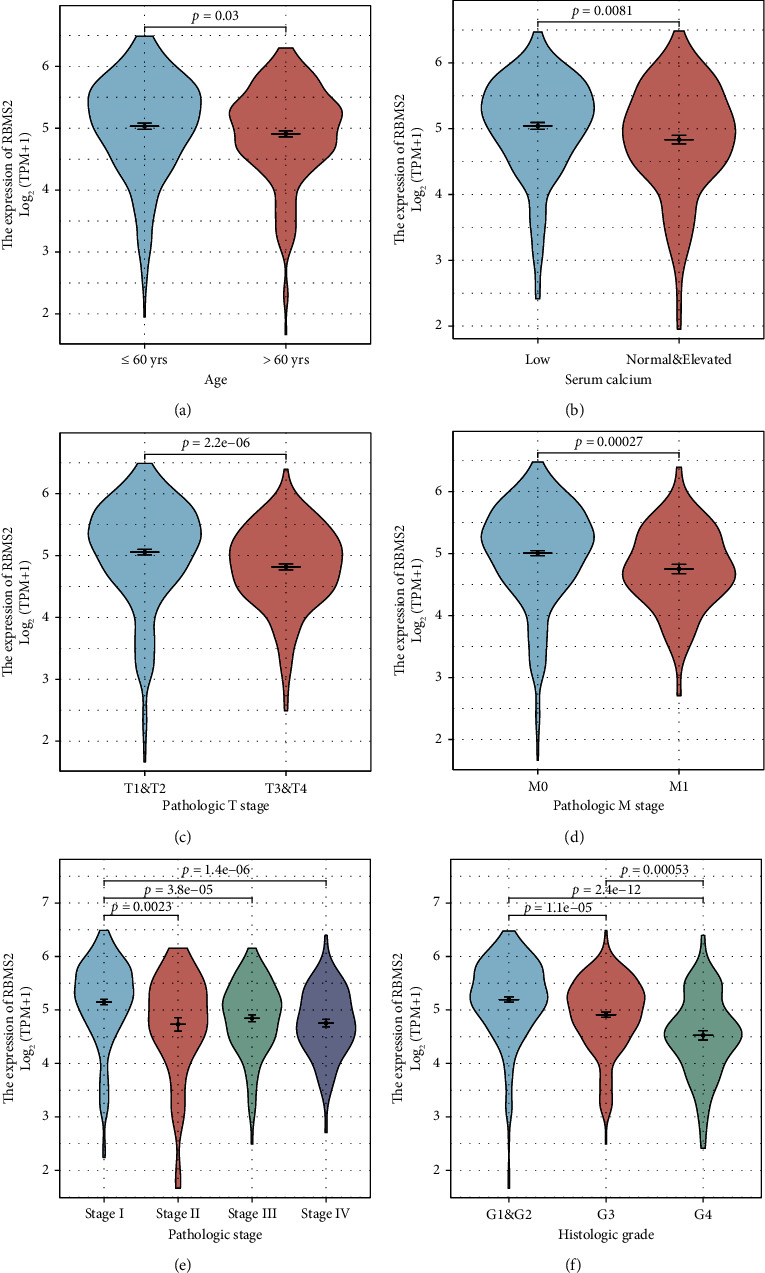
Associations between RBMS2 and clinico-pathological traits of ccRCC patients. RBMS2 mRNA levels were found to be significantly diminished in ccRCC patients presenting with elder age (a), elevated serum calcium level (b), advanced T stage (c), distant metastasis (d), advanced TNM stage (e), and poorer differentiation grade (f).

Figure 1 and Table 1 showcase a significant downregulation of RBMS2 expression in ccRCC patients presenting with severe disease markers, such as advanced age (*P* = 0.035), increased serum calcium level (*P* = 0.002), advanced T stage (*P* < 0.001), distant metastasis (*P* < 0.001), advanced TNM stage (*P* < 0.001), and poorer differentiation grade (*P* < 0.001).

**Figure 2:**
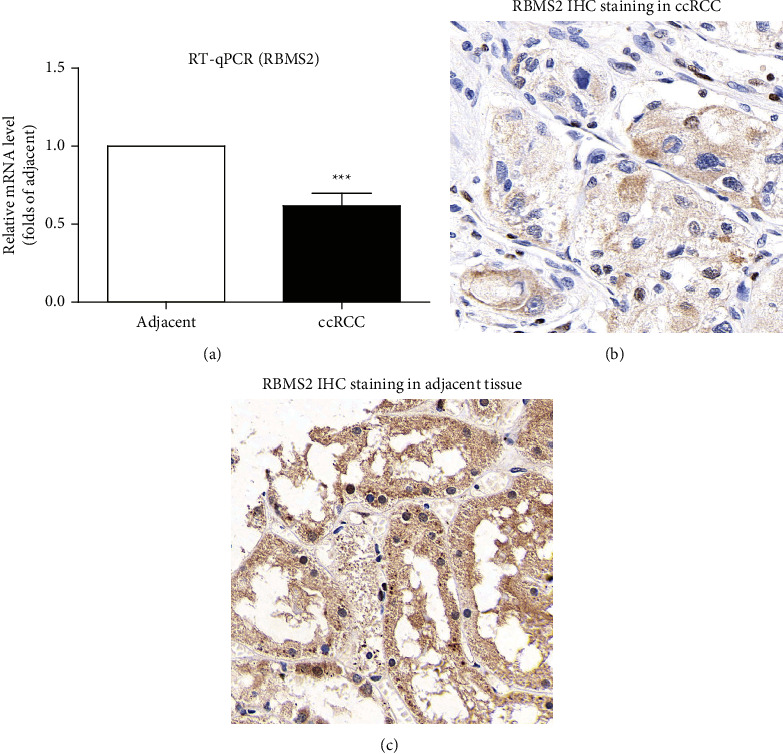
The mRNA and protein levels of RBMS2 in ccRCC specimens compared to adjacent nontumorous tissues. (a) RT-qPCR data reflected a decreased mRNA level of RBMS2 in ccRCC specimens compared to paired adjacent nontumorous tissues (*n* = 13, *P* = 0.0004). Data were compared by paired Student's *t*-test. (b) Representative IHC staining of RBMS2 protein in ccRCC tissues. (c) Representative IHC staining of RBMS2 protein in adjacent nontumorous tissues.

To further validate the expression difference of RBMS2 in ccRCC and adjacent nontumorous kidney tissues, we next collected 13 ccRCC specimens together with their paired nontumorous tissues. RT-qPCR data revealed that 10 of the 13 cases showed higher RBMS2 mRNA level in adjacent tissues than that in ccRCC tissues, and the mean fold change in each tissue pair is exhibited in Figure 2(a). Consistently, IHC immunostaining also reflected higher protein level of RBMS2 in nontumorous kidney tissue than that in ccRCC tissue (Figures 2(b) and 2(c)).

### 5.2. The Prognostic Relevance of RBMS2 in ccRCC Patients

**Figure 3:**
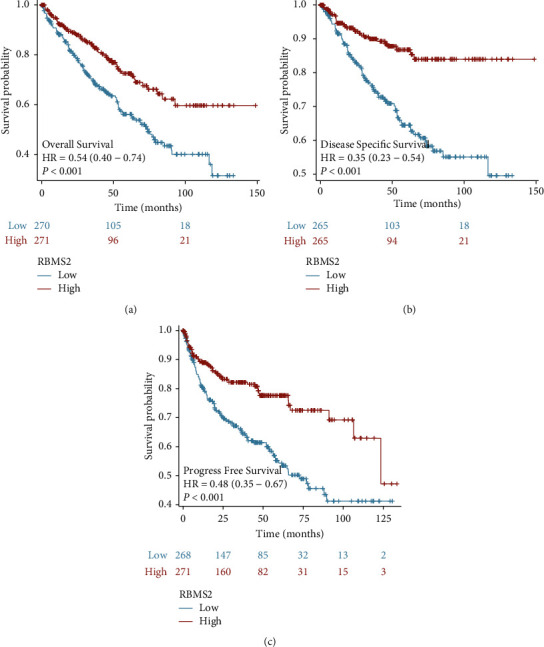
Prognostic relevance of RBMS2 in the ccRCC cohort. Higher RBMS2 levels correlated to improved overall survival (a), disease-specific survival (b), and progression-free survival (c), as depicted by the Kaplan–Meier analysis of the cohort from the TCGA dataset.

The prognostic potential of RBMS2 in the ccRCC cohort from the TCGA dataset is depicted in Figure 3. Downregulated RBMS2 is significantly correlated with worse overall survival (Figure 3(a)), disease-specific survival (Figure 3(b)), and progression-free survival (Figure 3(c)).

In the multivariate analysis for overall survival (Table 2), age over 60 years (hazard ratio = 1.728, *P* = 0.033), advanced T stage (hazard ratio = 2.207, *P* = 0.003), and the presence of metastasis (hazard ratio = 5.154, *P* < 0.001) were identified as factors significantly linked with worse survival. High RBMS2 expression was found to be a significant factor in the overall survival multivariate analysis although it did not reach statistical significance (hazard ratio = 0.719, *P* = 0.195). Similar trends were observed in the multivariate analysis for disease-specific survival (Table 3) and progression-free survival (Table 4), with advanced T stage and metastasis significantly associated with worse survival. Of note, high RBMS2 expression was significantly associated with better disease-specific survival (hazard ratio = 0.387, *P* = 0.004) and progression-free survival (hazard ratio = 0.527, *P* = 0.02).

### 5.3. Survival Nomograms for ccRCC Patients

**Figure 4:**
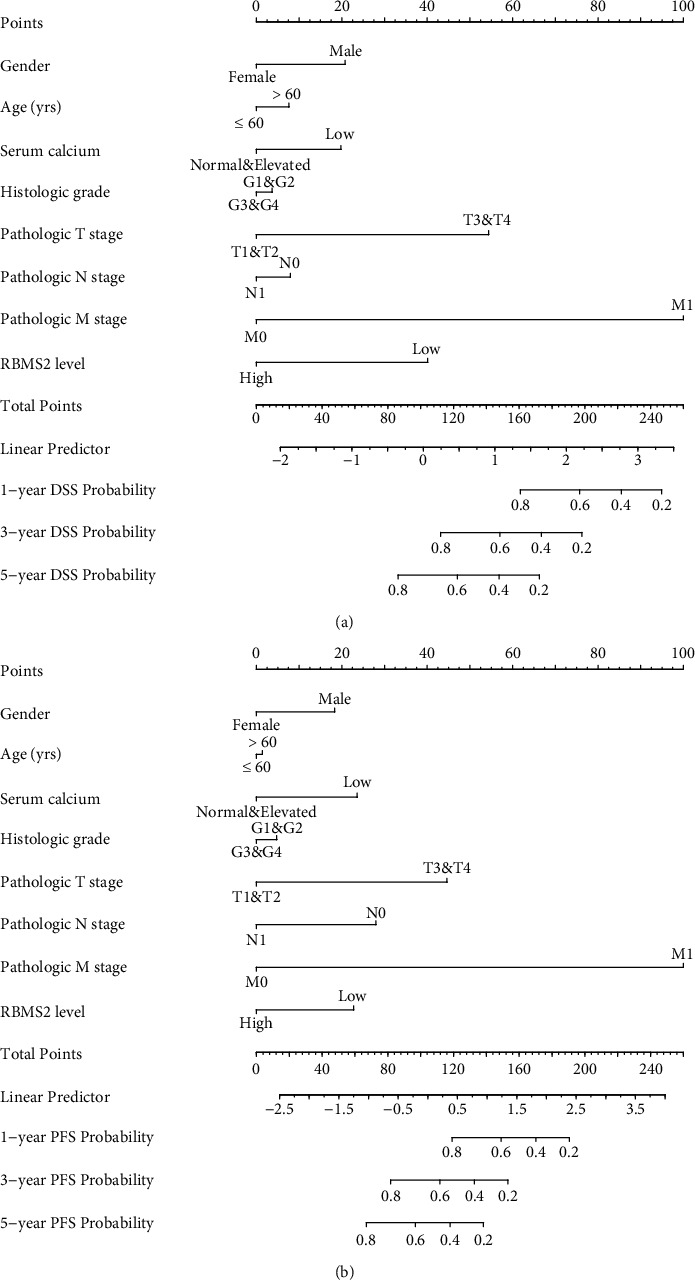
Survival nomograms for ccRCC patients. Disease-specific survival (a) and progression-free survival (b) nomograms were established to predict the 1-, 3-, and 5-year survival rates using the ccRCC cohort from TCGA dataset. The variables included are gender, age, serum calcium level, histological grade, T stage, N stage, M stage, and RBMS2 levels.

Based on the multivariate analyses, we next formulated disease-specific survival and progression-free survival nomograms to predict the 1-, 3-, and 5-year survival rates of ccRCC patients (Figures 4(a) and 4(b)). These nomograms incorporate various factors, including gender, age, serum calcium level, histological grade, T stage, N stage, M stage, and RBMS2 levels.

### 5.4. Cellular Experiments on the Tumor-Suppressing Role of RBMS2 in ccRCC

**Figure 5:**
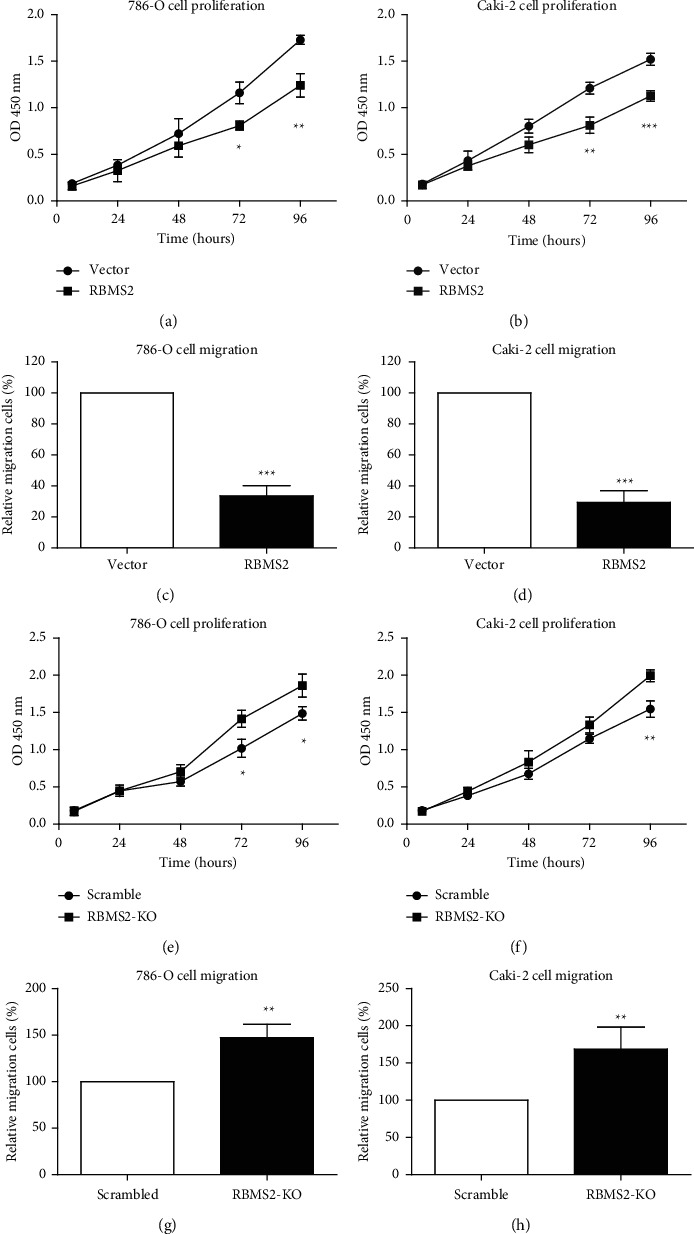
Cellular experiments examining the tumor-suppressing role of RBMS2 in ccRCC. (a, b) The CCK-8 assay revealed that overexpressing RBMS2 can significantly inhibit the proliferation capacity of 786-O and Caki-2 ccRCC cell lines. (c, d) The transwell migration assay demonstrated that overexpressing RBMS2 can significantly reduce the migration capacity of 786-O and Caki-2 ccRCC cell lines. (e, f) The CCK-8 assay revealed that RBMS2 knockdown can significantly enhance the proliferation capacity of 786-O and Caki-2 ccRCC cell lines. (g, h) The transwell migration assay demonstrated that RBMS2 knockdown can significantly upregulate the migration capacity of 786-O and Caki-2 ccRCC cell lines.

Cellular experiments were undertaken to examine the tumor-suppressing role of RBMS2 in ccRCC. We conducted overexpression and knockdown of RBMS2 in two different ccRCC cell lines, 786-O and Caki-2. The CCK-8 assay results demonstrated that overexpressing RBMS2 significantly inhibited the proliferation capacity of both 786-O and Caki-2 ccRCC cell lines (Figures 5(a) and 5(b)). In addition, the transwell migration assay results showed that overexpressing RBMS2 significantly reduced the migration capacity of these ccRCC cell lines (Figures 5(c) and 5(d)). In contrast, compared with the scrambled control group, siRNA-induced knockdown of RBMS2 significantly downregulates the proliferation and migration of the two cell lines (Figures 5(e)–5(h)).

### 5.5. Associations between RBMS2 Expression and Immune Infiltrations of ccRCC

**Figure 6:**
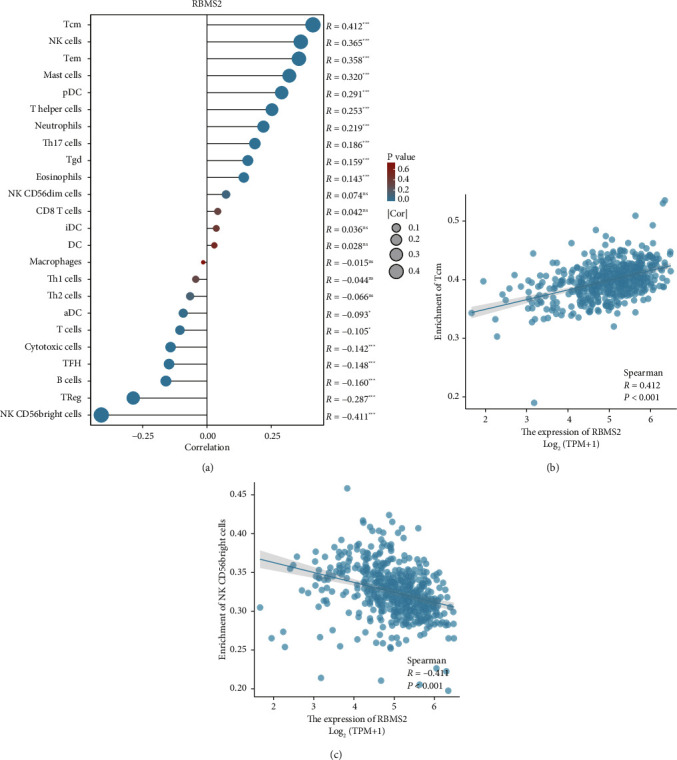
Correlations between RBMS2 expression and immune infiltrations in ccRCC. The enrichment of 24 immune cell types in samples with high or low RBMS2 levels was examined (a). For example, RBMS2 expression was positively correlated with Tcm cells (b) and negatively correlated with CD56bright cells (c) in ccRCC samples.

We also presented the correlations between RBMS2 expression and immune infiltrations in ccRCC.  Figure 6(a) shows different enrichments of 24 immune cell types in samples with high or low RBMS2 levels. Notably, RBMS2 expression showed a positive correlation with Tcm cells and a negative correlation with CD56bright cells in ccRCC samples (Figures 6(b) and 6(c)).

## 6. Discussion

Our research delves into the implications of RNA binding motif, single stranded interacting protein 2 (RBMS2) in kidney renal clear cell carcinoma (ccRCC), contributing to the growing body of evidence that underlines the tumor-suppressive role of RBMS2. Our findings reveal that RBMS2 expression is significantly downregulated in ccRCC patients with more severe disease characteristics. Moreover, RBMS2 has demonstrated potential as a prognostic indicator for survival outcomes in ccRCC.

Our findings are in line with previous research that has underscored the tumor-suppressive role of RBMS2 in various malignancies [2, 10]. RBMS2 is known to inhibit the activation of c-Myc, an oncogene that plays a pivotal role in cell proliferation and growth [2]. Downregulation of RBMS2 has been implicated in the pathogenesis and progression of various cancers, such as lung cancer and breast cancer. Our research extends these findings to ccRCC, suggesting a broad implication of RBMS2 in cancer biology. Furthermore, our in vitro experiments provide biological evidence for the tumor-suppressive role of RBMS2 in ccRCC. We have demonstrated that overexpression of RBMS2 can significantly inhibit the proliferation and migration of ccRCC cell lines. These findings highlight the therapeutic potential of RBMS2 in ccRCC. Indeed, RBMS2 was reported to chemosensitize breast cancer cells to doxorubicin, implying its therapeutic potential [3].

Additionally, our study has shed light on the interaction between RBMS2 expression and immune infiltration in the ccRCC tumor microenvironment, hinting at a role for RBMS2 in modulating the tumor immune microenvironment. The precise mechanisms through which RBMS2 influences immune infiltrations in ccRCC warrant further investigation.

While our study provides valuable insights, it does come with a few limitations. First, our findings are based on a retrospective analysis of a public database, and thus prospective studies are needed to validate these findings. Second, while our in vitro experiments have demonstrated the tumor-suppressive role of RBMS2 in ccRCC, further in vivo experiments and mechanistic studies are required to comprehensively understand the biological function of RBMS2 in ccRCC. Moving forward, our study opens several avenues for future research. Unraveling the molecular mechanisms underpinning the tumor-suppressive role of RBMS2 in ccRCC could provide novel insights into the pathogenesis of ccRCC. Additionally, as our study suggests a potential role of RBMS2 in modulating the tumor immune microenvironment, it would be worthwhile to investigate whether RBMS2 could serve as a target for immunotherapy.

## 7. Conclusions

Our research delves deep into the role of RBMS2 in ccRCC and reveals that RBMS2 expression is significantly diminished in patients displaying severe disease characteristics. Our findings suggest that higher RBMS2 expression levels correlate with improved survival outcomes, underscoring its potential as a prognostic biomarker. In vitro experiments further reinforce the tumor-inhibitory role of RBMS2 in ccRCC. Importantly, we have also uncovered correlations between RBMS2 expression and immune infiltration in ccRCC, suggesting a potential role of RBMS2 in modulating the tumor immune microenvironment. In conclusion, our study positions RBMS2 as a promising prognostic biomarker and a potential therapeutic target in ccRCC.

## Figures and Tables

**Table 1:** ccRCC patients' characteristics and correlations with RBMS2 expression level.

Characteristics	Low expression of RBMS2	High expression of RBMS2	*P* value
*n*	270	271	
Gender, *n* (%)			0.092
Female	84 (15.5%)	103 (19%)	
Male	186 (34.4%)	168 (31.1%)	
Age, *n* (%)			0.035
≤60	122 (22.6%)	147 (27.2%)	
>60	148 (27.4%)	124 (22.9%)	
Serum calcium, *n* (%)			0.002
Low	90 (24.5%)	114 (31.1%)	
Normal and elevated	98 (26.7%)	65 (17.7%)	
Histologic grade, *n* (%)			<0.001
G1 and G2	96 (18%)	154 (28.9%)	
G3 and G4	168 (31.5%)	115 (21.6%)	
Pathologic T stage, *n* (%)			<0.001
T1 and T2	150 (27.7%)	200 (37%)	
T3 and T4	120 (22.2%)	71 (13.1%)	
Pathologic N stage, *n* (%)			0.400
N0	125 (48.4%)	117 (45.3%)	
N1	10 (3.9%)	6 (2.3%)	
Pathologic M stage, *n* (%)			<0.001
M0	201 (39.6%)	228 (44.9%)	
M1	53 (10.4%)	26 (5.1%)	
Pathologic stage, *n* (%)			<0.001
Stage I and stage II	138 (25.7%)	194 (36.1%)	
Stage III and stage IV	129 (24%)	77 (14.3%)	

**Table 2:** ccRCC overall survival analysis.

Characteristics	Total (*N*)	Univariate analysis		Multivariate analysis	
		Hazard ratio (95% CI)	*P* value	Hazard ratio (95% CI)	*P* value
Gender	541		0.614		
Female	187	Reference			
Male	354	0.924 (0.679–1.257)	0.613		
Age	541		**<0.001**		
≤60	269	Reference		Reference	
>60	272	1.791 (1.319–2.432)	**<0.001**	1.728 (1.046–2.854)	**0.033**
Serum calcium	367		0.076		
Low	204	Reference		Reference	
Normal and elevated	163	1.356 (0.969–1.899)	0.076	0.622 (0.380–1.018)	0.059
Histologic grade	533		**<0.001**		
G1 and G2	250	Reference		Reference	
G3 and G4	283	2.665 (1.898–3.743)	**<0.001**	0.985 (0.560–1.735)	0.960
Pathologic T stage	541		**<0.001**		
T1 and T2	350	Reference		Reference	
T3 and T4	191	3.210 (2.373–4.342)	**<0.001**	2.207 (1.304–3.737)	**0.003**
Pathologic N stage	258		**0.001**		
N0	242	Reference		Reference	
N1	16	3.422 (1.817–6.446)	**<0.001**	0.829 (0.308–2.232)	0.710
Pathologic M stage	508		**<0.001**		
M0	429	Reference		Reference	
M1	79	4.401 (3.226–6.002)	**<0.001**	5.154 (2.926–9.080)	**<0.001**
RBMS2	541		**<0.001**		
Low	270	Reference		Reference	
High	271	0.545 (0.399–0.744)	**<0.001**	0.719 (0.436–1.184)	0.195

Bold values indicate *P*<0.05.

**Table 3:** ccRCC disease-free survival analysis.

Characteristics	Total (*N*)	Univariate analysis		Multivariate analysis	
		Hazard ratio (95% CI)	*P* value	Hazard ratio (95% CI)	*P* value
Gender	530		0.415		
Female	181	Reference			
Male	349	1.183 (0.786–1.781)	0.420		
Age	530		0.117		
≤60	265	Reference			
>60	265	1.351 (0.926–1.971)	0.118		
Serum calcium	361		0.056		
Low	200	Reference		Reference	
Normal and elevated	161	1.507 (0.989–2.298)	0.057	0.570 (0.312–1.041)	0.067
Histologic grade	522		**<0.001**		
G1 and G2	249	Reference		Reference	
G3 and G4	273	4.850 (2.925–8.043)	**<0.001**	0.936 (0.429–2.039)	0.867
Pathologic T stage	530		**<0.001**		
T1 and T2	347	Reference		Reference	
T3 and T4	183	5.606 (3.697–8.502)	**<0.001**	3.590 (1.752–7.358)	**<0.001**
Pathologic N stage	256		**0.003**		
N0	241	Reference		Reference	
N1	15	3.864 (1.831–8.157)	**<0.001**	0.764 (0.274–2.124)	0.605
Pathologic M stage	497		**<0.001**		
M0	422	Reference		Reference	
M1	75	9.219 (6.294–13.504)	**<0.001**	9.294 (4.797–18.004)	**<0.001**
RBMS2	530		**<0.001**		
Low	265	Reference		Reference	
High	265	0.352 (0.229–0.540)	**<0.001**	0.387 (0.203–0.737)	**0.004**

Bold values indicate *P*<0.05.

**Table 4:** ccRCC progression-free survival.

Characteristics	Total (*N*)	Univariate analysis		Multivariate analysis	
		Hazard ratio (95% CI)	*P* value	Hazard ratio (95% CI)	*P* value
Gender	539		**0.024**		
Female	186	Reference		Reference	
Male	353	1.476 (1.043–2.090)	**0.028**	1.650 (0.980–2.779)	0.060
Age	539		0.113		
≤60	268	Reference			
>60	271	1.285 (0.942–1.754)	0.114		
Serum calcium	366		0.054		
Low	203	Reference		Reference	
Normal and elevated	163	1.422 (0.994–2.034)	0.054	0.520 (0.301–0.901)	**0.020**
Histologic grade	531		**<0.001**		
G1 and G2	250	Reference		Reference	
G3 and G4	281	3.684 (2.530–5.364)	**<0.001**	0.873 (0.465–1.639)	0.672
Pathologic T stage	539		**<0.001**		
T1 and T2	350	Reference		Reference	
T3 and T4	189	4.569 (3.306–6.314)	**<0.001**	3.443 (1.923–6.167)	**< 0.001**
Pathologic N stage	257		**0.001**		
N0	241	Reference		Reference	
N1	16	3.697 (1.899–7.198)	**<0.001**	0.461 (0.166–1.276)	0.136
Pathologic M stage	506		**<0.001**		
M0	429	Reference		Reference	
M1	77	9.081 (6.554–12.582)	**<0.001**	15.996 (7.983–32.049)	**< 0.001**
RBMS2	539		**<0.001**		
Low	268	Reference		Reference	
High	271	0.481 (0.347–0.668)	**<0.001**	0.527 (0.308–0.903)	**0.020**

Bold values indicate *P*<0.05.

## Data Availability

The data used to support the findings of this study are available from the corresponding author upon request.
